# Surgical Cases Occurring in the Practice of a Country Doctor

**Published:** 1898-04

**Authors:** J. Frank Thornton

**Affiliations:** Plum, Texas


For the Texas Medical Journal.

### Surgical Cases Occurring in the Practice of a Country Doctor.

BY J. FRANK THORNTON, A. M., M. D., PLUM, TEXAS.

Feeling that it may be profitable to the young M. D. to review work done in the past, and knowing that reliable statistics can be obtained in only one way, and that is to report both the favorable and unfavorable cases, I will venture to call your attention to a few of my most important surgical cases treated during the past year. A review of such cases should prove profitable to the country practitioner, since we can never know when an emergency may be forced upon us. I will state here, that "cleanliness is next to Godliness," but I conduct it on the general principles of antiseptic surgery, ignoring the minute details. First of all, I cleanse the parts with carbolized water. My instruments are cleaned and freed from vaseline, then boiled. My sponges are cleaned in a bichloride solution 1 to 2000. When the operation is finished, I wait awhile, if possible, to see that there is no hemorrhage before applying my dressings; then if there is hemorrhage, it can be easily checked. When ready to unite the edges, I generally use a Hagerdorn needle and iron dyed silk. Then I apply over the closed edges iodoform gauze, after thoroughly washing the wound with a bichloride solution 1 to 2000. Then dry cotton is applied and held in place by a strip of adhesive plaster or a bandage. I never trouble my dressings if I can avoid it, but if suppurations ensue, then it has to be changed. I then cleanse the wound as at first, and dress it. In this way I have had good success in antiseptic surgery. I have not seen many cases of poetical surgery, for in nearly all of my cases pus formed, and there was an elevation of the temperature, but in the milder cases it was not so bad.

I wish to invite your attention to a brief report of the cases upon which I operated during the past year:

March 4, 1897. Mr. J., colored; age, 50 years; acute gangrene of the leg; due to embolism. When I saw him the line of demarkation was established, and there was only one thing to be done; the leg was amputated at the upper third. Reaction slight. There was a tendency of the posterior flap to slough. He was put on tonics, and with careful nursing he made a rapid recovery, being up and well in six weeks.

June 7, 1897, I was called to see Mr. J. C., who had been shot in the arm. The arm was amputated two inches below shoulder. There was much laceration of the muscular tissues, also a longitudinal fracture of the humerus. I first saw this man two days after he had been shot. There was an offensive odor which permeated his house. Maggots were here, and plenty of them. After the operation there was profound shock. I gave nitroglycerine hypodermically. Wound was cleansed with carbolic acid solution and dressed with iodoform gauze. Cleanliness and a nutritious diet were looked after. In three weeks patient was up and well.

September 5, 1897. Mr. H., white, age 40 years, arm caught in gin saws and torn almost off. I amputated below the shoulder joint. Made a circular cut and a longitudinal incision along the outer side of arm, and carried the sleeve of skin up the humerus. Perfect drainage was secured. There was a tendency to slough. There were also a number of spiculæ of bone discharged. The stump has pained him every now and then; recovery very slow.

November 8, 1897. Mr. C., age 22 years, while roping a steer had his little finger torn nearly off by the rope. I amputated the finger. The adjacent tissues were burnt by friction, and there was a great deal of suppuration, but complete recovery.

November 21, 1897. Mr. T., age 36 years, had been shot in the forearm with a double-barrel shotgun. About one-third of the muscular tissue on the anterior aspect of the arm had been torn away by the discharge of bird shot. Fortunately neither vessels nor bones were injured. The torn shreds of skin and muscular fibres were clipped off, and the wound was washed with carbolic acid solution, and then dressed with carbolized gauze. Suppuration ensued, but he made a rapid recovery, and now has a useful arm.

December 25, 1897. Mrs. W., age 42 years. Upon examination I found carcinoma of mammary gland. I informed her that an operation was necessary, to which she gave her consent. She was placed on a table with her chest elevated. The arm was held at right angles by my assistant. The integument was thoroughly cleansed with a 1 to 2000 sublimate solution, and shaved. Then I placed sublimate towels over the exposed surface, leaving the part to be removed in sight. My incision was made about two inches from the induration, and the skin and subcutaneous tissues divided down to the muscles. Then I dissected up the fascia covering the thoracic muscles with the gland. All vessels were tied with catgut as fast as they were divided. Oozing of blood was profuse, but was checked by pressing sponges into the wound. Ths thoracic artery was not divided until the tumor had been lifted up and completely severed. I had to dissect the adipose tissue along the gland which was enlarged. I had perfect drainage from the deepest portion of the wound. Then I closed the opening as much as possible, with sutures. After final irrigation with 1 to 3000 sublimate solution, I applied a loose dressing; there was protection placed over this dressing. Shock was very small, did not amount to anything. Patient rallied well and made a complete recovery. There has been no sign of the return of the malignant growth.

I hope the country practitioners will all record their surgical cases, and the review will prove profitable to many. Mine have been of great assistance to me.

